# Intestinal Microbiota Composition of Interleukin-10 Deficient C57BL/6J Mice and Susceptibility to *Helicobacter hepaticus*-Induced Colitis

**DOI:** 10.1371/journal.pone.0070783

**Published:** 2013-08-09

**Authors:** Ines Yang, Daniel Eibach, Friederike Kops, Birgit Brenneke, Sabrina Woltemate, Jessika Schulze, André Bleich, Achim D. Gruber, Sureshkumar Muthupalani, James G. Fox, Christine Josenhans, Sebastian Suerbaum

**Affiliations:** 1 Institute of Medical Microbiology and Hospital Epidemiology, Hannover Medical School, Hannover, Germany; 2 DZIF – German Center for Infection Research, Hannover-Braunschweig Site, Hannover, Germany; 3 Institute for Laboratory Animal Science, Hannover Medical School, Hannover, Germany; 4 Institute of Veterinary Pathology, Free University Berlin, Berlin, Germany; 5 Division of Comparative Medicine, Massachusetts Institute of Technology, Cambridge, Massachusetts, United States of America; Charité, Campus Benjamin Franklin, Germany

## Abstract

The mouse pathobiont *Helicobacter hepaticus* can induce typhlocolitis in interleukin-10-deficient mice, and *H. hepaticus* infection of immunodeficient mice is widely used as a model to study the role of pathogens and commensal bacteria in the pathogenesis of inflammatory bowel disease. C57BL/6J *Il10^−/−^* mice kept under specific pathogen-free conditions in two different facilities (MHH and MIT), displayed strong differences with respect to their susceptibilities to *H. hepaticus*-induced intestinal pathology. Mice at MIT developed robust typhlocolitis after infection with *H. hepaticus*, while mice at MHH developed no significant pathology after infection with the same *H. hepaticus* strain. We hypothesized that the intestinal microbiota might be responsible for these differences and therefore performed high resolution analysis of the intestinal microbiota composition in uninfected mice from the two facilities by deep sequencing of partial 16S rRNA amplicons. The microbiota composition differed markedly between mice from both facilities. Significant differences were also detected between two groups of MHH mice born in different years. Of the 119 operational taxonomic units (OTUs) that occurred in at least half the cecum or colon samples of at least one mouse group, 24 were only found in MIT mice, and another 13 OTUs could only be found in MHH samples. While most of the MHH-specific OTUs could only be identified to class or family level, the MIT-specific set contained OTUs identified to genus or species level, including the opportunistic pathogen, *Bilophila wadsworthia*. The susceptibility to *H. hepaticus*-induced colitis differed considerably between *Il10^−/−^* mice originating from the two institutions. This was associated with significant differences in microbiota composition, highlighting the importance of characterizing the intestinal microbiome when studying murine models of IBD.

## Introduction

The gram-negative ‘pathobiont’ *Helicobacter hepaticus* primarily colonizes the gastrointestinal tract of mice, and can also colonize bile canaliculi and gallbladder of mice with chronic hepatitis [1]. While *H. hepaticus* colonization causes no intestinal pathology in immunocompetent mice, it induces hepatobiliary inflammation and an increased incidence of hepatic cancer in a number of susceptible mouse strains [2]–[4]. In mice with compromised immune function, *H. hepaticus* in combination with other constituents of the murine intestinal microbiota can induce inflammatory bowel disease (IBD) [5]–[7]. *H. hepaticus* infections in immunodeficient mice with or without transfer of T cells have been widely used as model systems for human IBD ([5], [8]–[10],for a review see [11]). The *H. hepaticus* infected *Rag* ^−/−^ mouse model is also widely used as a model of microbially driven colon cancer [12], [13].

Induction of intestinal inflammation in mice depends on a number of known *H. hepaticus* virulence factors. In addition to traits critical for successful colonization, such as flagellar motility [14], a number of genes and mechanisms directly influencing pathology have been identified. The *H. hepaticus* genome [15] contains a gene cluster encoding a cytolethal distending toxin (CDT), a homolog of the *Campylobacter jejuni* CDT, which causes cell cycle arrest, chromatin fragmentation, and apoptosis [16]. It also includes a pathogenicity island HHGI1, which encodes a functional type VI secretion system [17], [18]. Strains carrying the island had a higher potential to induce hepatitis in A/JCr mice [19]. Mutants lacking parts of the HHGI1 island were attenuated with respect to the induction of typhlocolitis in *Il10^−/−^* mice [20]. *H. hepaticus* lipopolysaccharide (LPS) has been shown to reduce Toll-like receptor 4 (TLR4) and TLR5-mediated innate immune responses of intestinal epithelial cells, but also to inhibit development of endotoxin tolerance, thus affecting host responses to the resident microbiota and intestinal inflammatory conditions [21].

Infection with *H. hepaticus* alone is not sufficient to induce IBD in *Il10^−/−^* mice [22], [23]. IBD is induced only in the presence of normal murine microbiota, or during co-infection with specific other bacteria [11], [23]. The effect of the resulting inflammation on the intestinal microbiota depends both on the host immune response and on characteristics of the microbiota, both of which might vary between different mouse strains [7], [24].

An involvement of the intestinal microbiota in the etiology of IBD has been demonstrated in a variety of mouse models. Inflammation induced in response to bacterial infection, a chemical inducer or genetic factors can alter the composition of the intestinal microbiota, which can be accompanied by changes in overall bacterial abundance and diversity [11]. Both in hosts with compromised immune regulation [25] and in mice with deficiencies in colonic epithelial cell inflammasomes [26], these changes can transform the microbiota into a colitogenic state. If this colitogenic microbiota is transmitted to wild type (wt) animals, it can confer a predisposition for inflammation in these healthy hosts [25], [26].

In mouse models of IBD, such as in *H. hepaticus* infected *Il10^−/−^* mice, this dysbiosis and its interdependence with inflammatory processes can be studied in controlled settings. However, even in inbred strains of mice kept under specific pathogen-free (SPF) conditions, the exact composition of the intestinal microbiota is rarely known before the start of the experiment. This study started with the observation that C57BL/6J *Il10^−/−^* mice kept at two different animal facilities displayed strongly discordant susceptibilities to *H. hepaticus*-induced typhlocolitis. We hypothesized that the intestinal microbiota composition might be responsible for these different susceptibilities, and therefore investigated the microbiota compositions of uninfected mice from these two facilities with the aim to identify microbiota components whose differential presence might contribute to or protect the mice from IBD after infection with *H. hepaticus*. In order to reduce the effect of random differences between groups of mice on our conclusions, we included two groups of MHH mice that were born in different years.

## Results

### Differential Susceptibility to *H. hepaticus*-associated Typhlocolitis of C57BL/6J *Il10^−/−^* mice Housed in Two Different Animal Facilities

**Figure 1:**
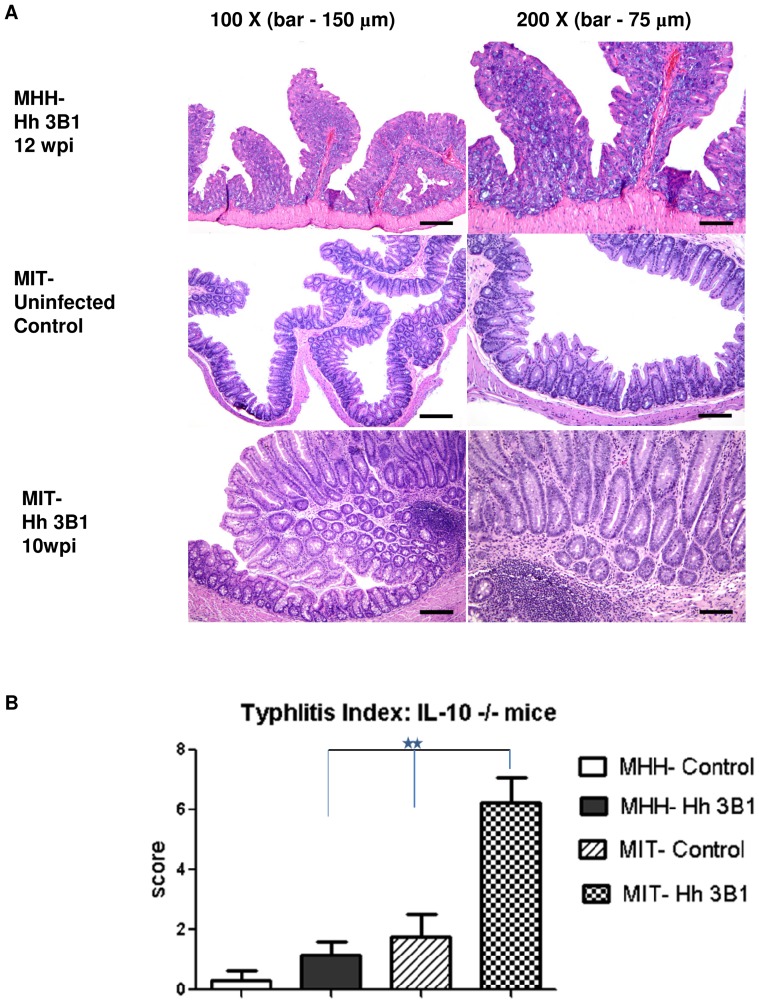
Effects of *H. hepaticus* infection on MIT vs. MHH mice. Intestinal inflammation induced by *H. hepaticus* infection in C57BL/6J *Il10^−/−^* mice reared in MIT and MHH SPF facilities. A, histological sections (H&E stain); B, Histological sections of cecum were scored separately for inflammation, epithelial defects, edema, crypt atrophy, hyperplasia and dysplasia on a scale of 0 to 4. The scores from all the different parameters were added and represented as Typhlitis Index scores as described in Burich *et al.* [29]. P values according to nonparametric Mann-Whitney test, **<0.005.

*H. hepaticus* infected C57BL/6 *Il10^−/−^* mice were reported to develop typhlocolitis by the group of J.G.F. at MIT [20], [27], and similar findings have been reported by other groups [6] [28] [29]. By contrast, attempts by the group of S.S. to establish this model at Hannover Medical School (MHH) with the same *H. hepaticus* strain, ATCC 51449 (3B1), following an identical protocol for inoculation and monitoring of infection, yielded different results. While mice at MIT developed robust typhlocolitis, mice at MHH reproducibly did not develop significant inflammation (Fig. 1), despite a high level colonization with *H. hepaticus* 3B1 (see Materials and Methods). A potential attenuation of the strain 3B1 due to laboratory passage was ruled out by transfer of a fresh infectious 3B1 isolate from MIT to MHH prior to the experiment depicted in Fig. 1. Furthermore, the strain 3B1 used at MHH caused robust inflammation comparable with results from other laboratories in different (e.g. T cell transfer) models of *H. hepaticus*-induced colitis [17]. We therefore hypothesized that differences of the intestinal microbiota composition between C57BL/6 *Il10^−/−^* mice kept at the two different facilities might be responsible for these observed major differences of susceptibility to *H. hepaticus*-induced pathology. We therefore performed a high resolution analysis of microbiota composition for C57BL/6J *Il10^−/−^* mice from both facilities.

### Microbiota Composition Analysis of C57BL/6J *Il10^−/−^* mice Reared at MIT or MHH

We analyzed the intestinal microbiota composition of three groups of *Helicobacter*-free mice from breeding colonies at MHH and MIT used for *H. hepaticus* infection experiments, such as the representative experiments described in the previous section. Each group consisted of eight C57BL/6J *Il10^−/−^* mice kept under SPF conditions (see Materials and Methods for details). Two of these groups of mice (MHH2009 and MHH2011) were housed at MHH and one group was housed at MIT. Microbiota composition analysis was performed by partial amplification of 16S rRNA genes and deep sequencing using 454 FLX technology (see Material and Methods for details).

We generated a total of 247,219 sequences, which were pre-processed using the mothur pipeline [30]. After a series of quality checks, which included removal of low-quality sequences and of PCR chimeras, 124,930 (51%) sequences were retained. The mean sequence length of the pre-processed sequences was 261 bases. Using the RDP classifier (Bootstrap cutoff 70%) [31], 114,994 of the 124,930 pre-processed sequences (92%) were identified at least to class level, or were assigned to one of the genera unclassified in the RDP dataset (“OD1” and TM7). The remaining sequences were excluded from subsequent analysis following Ochman *et al.* [32]. No mitochondrial or chloroplast sequences were found in the dataset.

Overall, 28,982 sequences (25% of all retained sequences) were identified to genus at bootstrap support (BTS) 70% (Table S1). Of these, 17,068 sequences (59%) could be further classified to species level using a MegaBlast approach and a modified RDP database. Those sequences were used to choose the distance level of 0.08 as the OTU set for which species and OTU boundaries best correspond (Fig. S1). At this level, the retained 114,994 sequences clustered into 287 OTUs. After subsampling the dataset to 1227 sequences per sample, which was done to ensure equal sequence numbers in all samples, 246 OTUs remained (Table 1 and Table S2). Good’s coverage, which measures the proportion of OTUs sampled at least twice, was 97.5% or higher for all individual samples, and 99.7% or higher for each sample set from one gut region of one group of mice. Of the 246 OTUs retained after subsampling, 119 occurred in at least half the cecum or colon samples of at least one mouse group; the other 127 were considered rare (Table S3). Of the 119 OTUs occurring in at least half the cecum or colon samples of at least one group of mice, 99 (83%) were identified as significantly differently prevalent between all MHH and MIT samples or at least one set of MHH samples and their MIT counterparts (Table S3).

**Table 1:** OTU counts and Good’s coverage (dataset containing 1227 sequences per sample, 8 samples per sample set).

Sample set	OTUs	Good’s coverage
**MIT_cecum**	163	99.70%
**MIT_colon**	173	99.70%
**MHH2009_cecum**	104	99.80%
**MHH2009_colon**	98	99.80%
**MHH2011_cecum**	114	99.80%
**whole dataset**	246	

### Comparisons between Sample Sets

**Figure 2:**
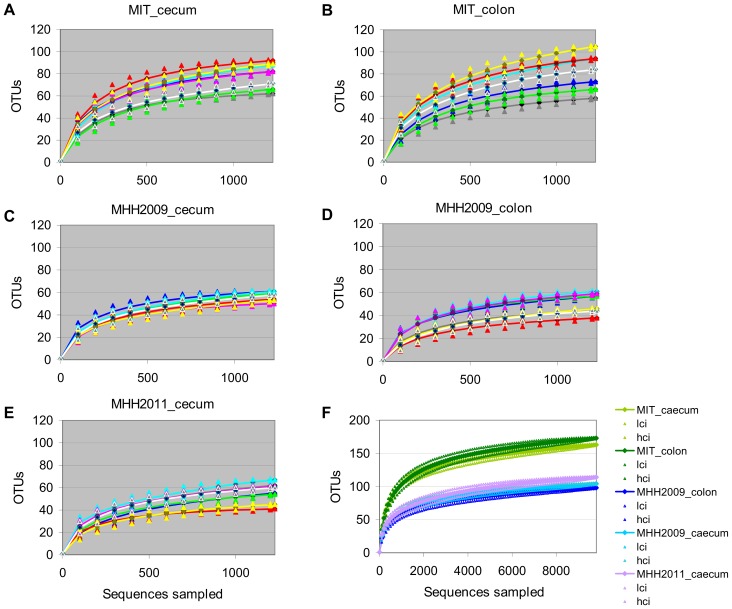
Rarefaction curves. A–E, rarefaction curves for individual samples (n = 8 mice), grouped by sample set. F, rarefaction curves for combined data for each sample set (n = 5 sets), including 95% confidence intervals. Lci, lower bound of confidence interval; hci, higher bound of confidence interval. Each sample set consist of either the cecum or the colon samples for one batch of 8 mice. All curves generated after subsampling to 1227 sequences per sample.

**Figure 3:**
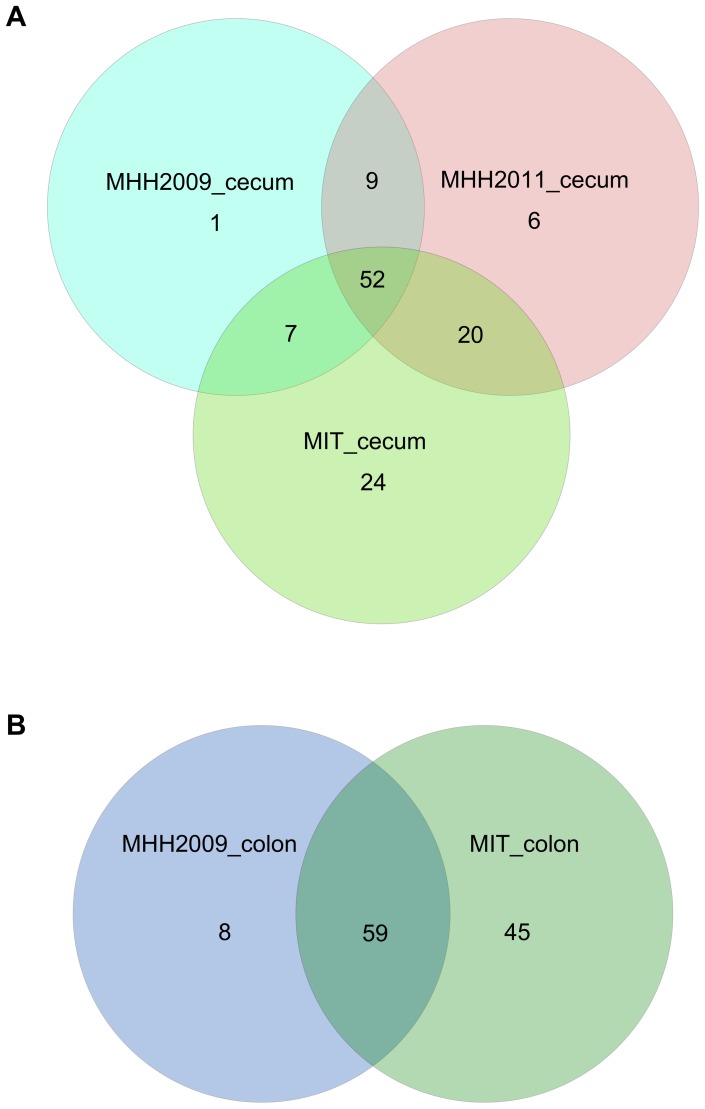
Distribution of OTUs occurring in at least 4 samples of the same set. A, cecum samples, B, colon samples.

**Figure 4:**
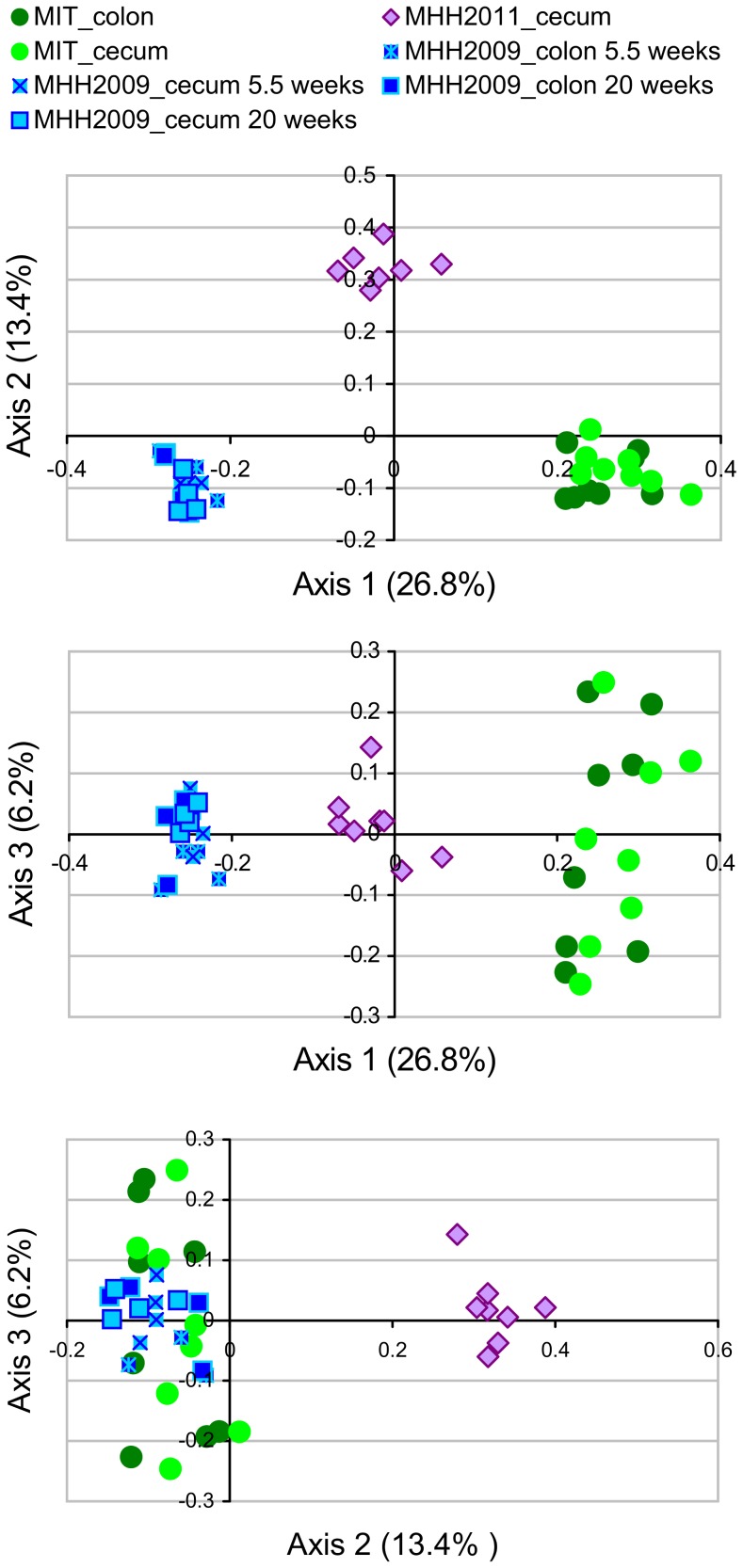
Principal coordinates analysis (PCoA) of microbiota samples. Based on Jaccard index distances measuring dissimilarity of OTU-level microbiota composition between individual samples. Axis labels including fractions of variance explained.

The microbiota richness observed after subsampling to 1227 sequences per sample varied both between mice within one group (Fig. 2A–E) and between mouse groups (Fig. 2F). MIT mice contained considerably more OTUs than MHH animals. Microbiota composition differed between all groups of mice (Fig. 3). In a Principal Coordinates Analysis (PCoA) based on dissimilarity of microbiota compositions, the first two axes, which represented 26.8% and 13.4% of the variance in the dataset, grouped the samples according to mouse group (Fig. 4). While the first axis depicted MHH2011 samples as intermediate between MHH2009 and MIT samples, the second axis emphasized the difference between the MHH2011 and all other mice. The cecum and the colon sample sets of each mouse group were grouped together. Axes 3 to 14, which represented 6.2% to 1.7% of the variance, did not resolve the difference between mouse groups (axes 4 to 14 not shown). Removing the rare OTUs from the dataset did not appreciably change the PCoA results (data not shown).

**Figure 5:**
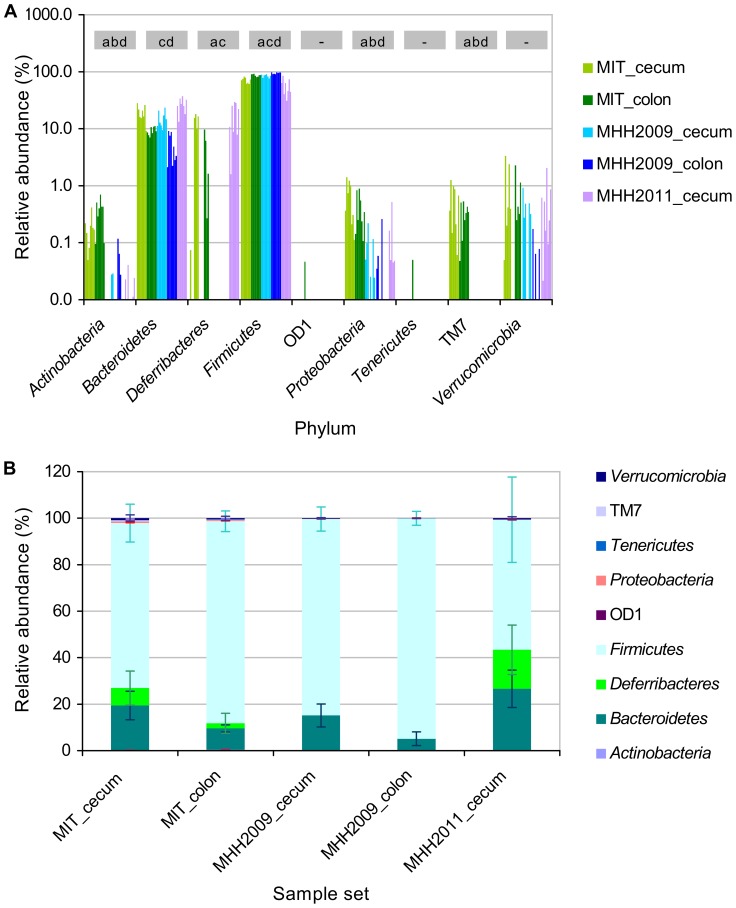
Phylum-level composition of microbiota. A. Fraction of sequence counts in each of the individual samples, color coded by sample group. Grey boxes indicate significant differences between sample groups (unequal variances t-Test, p<0.05): a, significantly different between MIT and MHH2009 cecum samples; b, significantly different between MIT and MHH2011 cecum samples; c, significantly different between MHH2009 and MHH2011 cecum samples; d, significantly different between MIT and MHH2009 colon samples. B. Relative abundance of phyla in each of the sample groups, including standard deviation (n = 8).

These differences between groups were reflected in the phylum-level composition of the microbiomes (Figure 5 A, B), which was determined by RDP classifier analysis of all 114,994 high-quality sequences. *Actinobacteria* and *Proteobacteria* were significantly more abundant in MIT than in MHH samples (two-sided unequal variances t-Test on relative sequence counts, p<0.05). Candidate division TM7, which was represented with only one OTU, was restricted to MIT mice, where it occurred in all samples (see Fig. 5 and Table S3). In MHH2009 samples, *Firmicutes* accounted for a significantly higher percentage of sequences of both cecum and colon samples than in the two other mouse groups. The difference between MIT and MHH2011 cecum samples was not significant at p≤0.05. *Bacteroidetes* were significantly less abundant in MHH2009 than in MHH2011 cecum samples, and in MHH2009 colon than in MIT colon samples.

**Figure 6:**
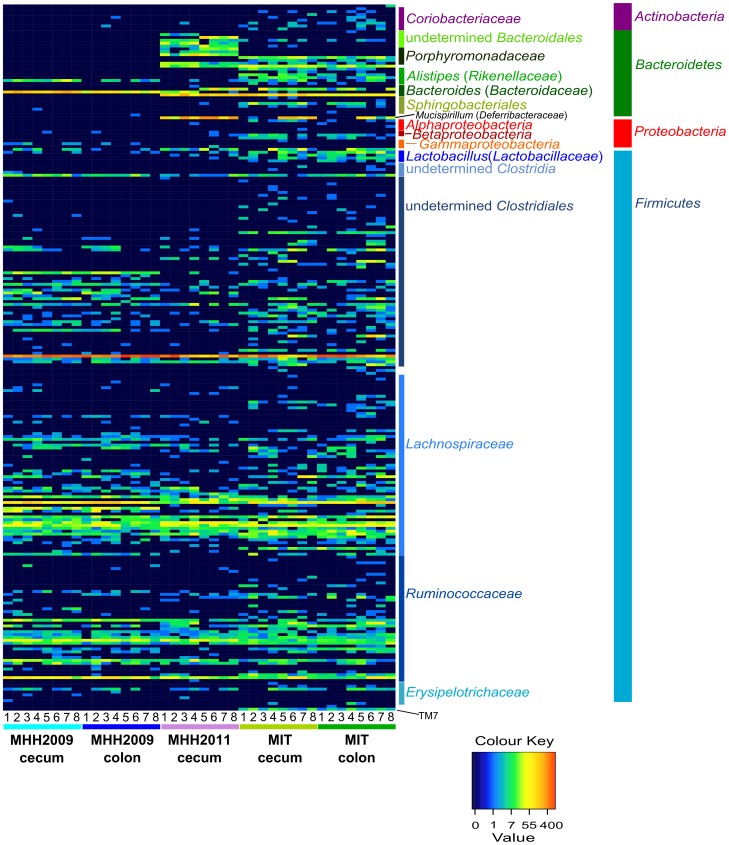
Distribution of OTUs among samples. Colors encode absolute OTU counts. Information on OTU classification according to RDP classifier. Numbers 1–8 are mouse identifiers. Within one mouse batch, equal numbers refer to cecum and colon samples of the same animal. Heatmap generated after subsampling to 1227 sequences per sample. The numbers on the color key correspond to untransformed OTU abundances.

The distribution of *Deferribacteres*, which were represented by only one OTU classified as *Mucispirillum schaedleri*, varied considerably among the individual mice of the MIT and MHH2011 groups. They did not occur in MHH2009 mice (Fig. 6 and Table S3).

Of the 119 OTUs not classified as rare, 24 were only found in MIT samples (Table 2, Table S3). Of these, *Parabacteroides merdae*, *Lactobacillus intestinalis* and *Clostridium* XIVb belonged to genera also identified from the sequences of the altered Schaedler flora (ASF) as provided by Dewhirst *et al.* [33] (Table S4). The most abundant MIT-specific OTU was classified as *Odoribacter*, a member of the *Porphyromonadaceae* within the *Bacteroidales*. 13 of the 24 OTUs were members of the *Clostridiales*. Eight of these could be further classified as *Lachnospiraceae*, including the *Clostridium* XIVb OTU mentioned above. Four of the MIT-specific OTUs were members of the *Rikenellaceae* genus *Alistipes*. This genus was also represented by a rare OTU only found in MIT samples (but not included in the list of MIT-specific OTUs because it was only found in 3 mice) and a further OTU classified as *Alistipes finegoldii* that occurred in all mouse groups Other MIT-specific OTUs were classified as *Bilophila wadsworthia* (*Desulfovibrionaceae*), a member of the Sphingobacteriales, and a member of the candidate division TM7.

**Table 2:** MIT-specific OTUs occurring in at least 4 samples of one MIT sample set.

OTU label	OTU classification	Number of reads assigned to OTU(samples with OTU)	
		MIT, cecum	MIT, colon
0.08_group_254	*Bacteroidetes*, *Bacteroidia*, *Bacteroidales*, *Porphyromonadaceae*, *Odoribacter*	141 (8)	104 (8)
0.08_group_249	*Firmicutes*, *Clostridia*, *Clostridiales*	49 (3)	172 (4)
0.08_group_243	*Firmicutes*, *Clostridia*, *Clostridiales*, *Lachnospiraceae*, *Clostridium XlVb*	61 (7)	69 (8)
0.08_group_126	*Bacteroidetes*, *Bacteroidia*, *Bacteroidales*, *Rikenellaceae*, *Alistipes*	71 (7)	37 (7)
0.08_group_106	*Firmicutes*, *Clostridia*, *Clostridiales*, *Lachnospiraceae*	60 (7)	38 (7)
0.08_group_191	*TM7*, *genera*, *incertae sedis*	58 (8)	38 (8)
0.08_group_120	*Bacteroidetes*, *Bacteroidia*, *Bacteroidales*, *Rikenellaceae*, *Alistipes*	72 (6)	21 (5)
0.08_group_286	*Firmicutes*, *Clostridia*, *Clostridiales*	37 (4)	52 (3)
0.08_group_270	*Firmicutes*, *Clostridia*, *Clostridiales*	37 (7)	25 (5)
0.08_group_104	*Bacteroidetes*, *Bacteroidia*, *Bacteroidales*, *Rikenellaceae*, *Alistipes*	49 (8)	11 (5)
0.08_group_195	*Firmicutes*, *Clostridia*, *Clostridiales*, *Lachnospiraceae*	8 (4)	33 (6)
0.08_group_141	*Bacteroidetes*, *Bacteroidia*, *Bacteroidales*, *Porphyromonadaceae*, *Parabacteroides*, *Parabacteroides merdae*	22 (7)	15 (5)
0.08_group_131	*Proteobacteria*, *Deltaproteobacteria*, *Desulfovibrionales*, *Desulfovibrionaceae*,*Bilophila*, *Bilophila wadsworthia*	20 (6)	10 (4)
0.08_group_221	*Firmicutes*, *Clostridia*, *Clostridiales*	13 (6)	15 (5)
0.08_group_107	*Firmicutes*, *Clostridia*, *Clostridiales*, *Lachnospiraceae*	23 (6)	5 (2)
0.08_group_188	*Firmicutes*, *Clostridia*, *Clostridiales*, *Lachnospiraceae*	7 (3)	20 (4)
0.08_group_246	*Firmicutes*, *Bacilli*, *Lactobacillales*, *Lactobacillaceae*, *Lactobacillus*, *Lactobacillus intestinalis*	5 (3)	16 (5)
0.08_group_153	*Firmicutes*, *Clostridia*, *Clostridiales*, *Lachnospiraceae*	11 (4)	9 (4)
0.08_group_156	*Firmicutes*, *Clostridia*, *Clostridiales*	7 (4)	9 (5)
0.08_group_97	*Proteobacteria*, *Alphaproteobacteria*	13 (5)	3 (3)
0.08_group_119	*Firmicutes*, *Clostridia*, *Clostridiales*, *Lachnospiraceae*	9 (3)	7 (4)
0.08_group_144	*Bacteroidetes*, *Sphingobacteria*, *Sphingobacteriales*	6 (4)	6 (4)
0.08_group_118	*Firmicutes*, *Clostridia*, *Clostridiales*, *Lachnospiraceae*	2 (1)	9 (5)
0.08_group_260	*Bacteroidetes*, *Bacteroidia*, *Bacteroidales*, *Rikenellaceae*, *Alistipes*	9 (4)	1 (1)

No conclusive pattern could be detected among the MHH-specific OTUs (Table 3), as all OTUs not occurring in MIT mice were either non-existent in one of the MHH groups, or occurred in less than half of the samples and were thus classified as rare in either MHH2009 or MHH2011 mice (Fig. 6, see Table S3).

**Table 3:** MHH-specific OTUs occurring in at least 4 samples of one MHH sample set.

OTU label	OTU classification	Number of reads assigned to OTU (samples with OTU)		
		MHH2009, cecum	MHH2009, colon	MHH2011, cecum
0.08_group_252	*Firmicutes, Clostridia, Clostridiales, Lachnospiraceae*	224 (8)	222 (8)	5 (2)
0.08_group_169	*Firmicutes, Clostridia, Clostridiales*	116 (8)	127 (8)	7 (2)
0.08_group_199	*Firmicutes, Clostridia, Clostridiales, Lachnospiraceae*	64 (4)	50 (4)	36 (3)
0.08_group_130	*Firmicutes, Clostridia, Clostridiales*	24 (4)	24 (4)	6 (3)
0.08_group_121	*Firmicutes, Clostridia, Clostridiales, Ruminococcaceae*	13 (5)	9 (4)	2 (2)
0.08_group_117	*Proteobacteria, Gammaproteobacteria, Pseudomonadales, Moraxellaceae, Acinetobacter*	10 (5)	3 (2)	0 (0)
0.08_group_234	*Firmicutes, Clostridia, Clostridiales, Lachnospiraceae*	1 (1)	1 (1)	35 (7)
0.08_group_155	*Bacteroidetes, Bacteroidia, Bacteroidales*	0 (0)	0 (0)	18 (4)
0.08_group_170	*Bacteroidetes, Bacteroidia, Bacteroidales, Porphyromonadaceae*	0 (0)	0 (0)	36 (7)
0.08_group_157	*Bacteroidetes, Bacteroidia, Bacteroidales, Porphyromonadaceae*	0 (0)	0 (0)	79 (6)
0.08_group_198	*Bacteroidetes, Bacteroidia, Bacteroidales*	0 (0)	0 (0)	123 (7)
0.08_group_168	*Bacteroidetes, Bacteroidia, Bacteroidales*	0 (0)	0 (0)	244 (4)
0.08_group_184	*Bacteroidetes, Bacteroidia, Bacteroidales, Porphyromonadaceae*	0 (0)	0 (0)	317 (8)

## Discussion

*H. hepaticus*-infected immunodeficient mice are a widely used model system for the investigation of inflammatory bowel diseases [12]. While monoassociation with *H. hepaticus* has been reported to cause sporadic colitis in mono-associated Swiss Webster mice during very long-term infection [3], that does not normally hold true for the widely used *Il10^−/−^* mice [7], [22]. Experiments using the *Il10^−/−^ H. hepaticus*-induced colitis model are usually conducted in the presence of a normal mouse microbiota or SPF microbiota [20], [28], [29]; the exact composition of which remains mostly unknown. In spite of the widespread use of this model, different outcomes of *H. hepaticus* infection in *Il10^−/−^* mice are well known [6], [22]. Here we compared the microbiota of C57BL/6 *Il10* ^−/−^ mice that were reared at two different institutions and showed highly reproducible differences in their susceptibility to *H. hepaticus-*induced pathology.

We found major differences between the microbiota of the mice housed in the two different animal facilities. There was also significant (but much lower) microbiota variation between the two groups of MHH mice (Fig. 4), which had originated from the same breeding colony but were born in different years. Multiple factors may have contributed to the differences between the microbiota of the three mouse groups. These include differences in animal husbandry (e.g. chow, cohousing with other mouse groups) and the propagation of random effects during the seeding and development of the individual microbiota. In a study mainly searching for genetic factors influencing microbiota in mice, Benson *et al.* [34] also found significant effects of the litter of origin, even when accounting for the identity of the mother. Hufeldt *et al.* [35] detected significant differences between the cecal microbiota of mouse litters by dams of the same breeding colony, but significantly less variation if these dams were siblings from the same litter. Finally, minor genetic differences between the two colonies of mice which have been bred for several years at different facilities cannot be excluded and may also contribute to differences of microbiota composition.

Age has been reported as a determinant of microbiota composition in mice [36], [37]. The MHH2009 group contained mice of two different ages. An AMOVA analysis performed with mothur 1.27.0 found significant differences of microbiota composition between two subgroups of the MHH2009 groups that were aged 1 vs. 4 months (data not shown). However, this age effect was minor compared to the differences between MHH-raised and MIT-raised mice. The PCoA clustering of the same data confirmed that the effects of age were relatively minor (Fig. 4) while the differences between MHH and MIT-reared mice were substantial. This is consistent with the observation that the absence of *H. hepaticus*-induced pathology was reproducibly observed at the MHH facility when C57BL/6 *Il10^−/−^* mice of different ages (10–17 weeks at the time of first infection) were infected with *H. hepaticus* 3B1 (data not shown).

In the current study, colon and cecum samples clustered together for both MIT and MHH2009 mice (Fig. 4). This effect is also known from humans, where the microbiota between cecal and colonic mucosal samples from the same study participant tend to be rather similar [38], while considerable differences exist between individuals [38], [39].

In order to identify intestinal bacterial candidates that might either contribute to colitis development in MIT mice or protect the MHH mice from intestinal inflammation [40], [41], we compared the intestinal microbiota of both groups of MHH mice to their counterparts from the MIT.

Surprisingly, all MHH-specific (approximately species-level) OTUs were either not detected or classified as rare in at least one of the sample sets. We could however identify five OTUs that occurred in all groups of mice and that were significantly more abundant in MHH mice than in MIT mice (Table S3). In addition to the three *Lachnospiraceae* OTUs classified only to family level; the OTUs included *Bacteroides intestinalis* and an OTU of the genus *Oscillibacter*, which are both constituents of a healthy gut microbiota in humans [42], [43]. Notably, another *Oscillibacter* OTU was significantly more abundant in the corresponding MIT samples.

Of the group of 24 OTUs found to be MIT-specific, several were classified as taxa that are associated with healthy microbiota in humans, such as *Odoribacter*, *Parabacteroides merdae*, and *Alistipes* [42]–[45]. One OTU was identified as *Clostridium XlVb*, a taxon also identified from altered Schaedler flora (ASF) sequences (Table S4), implying that the corresponding organism is closely related to strain ASF 356 in the widely used defined 8-strain rodent ASF [33].

MIT-specific OTUs also included the one OTU of candidate division TM7 found in our dataset. Certain phylotypes within this lineage are associated with IBD in humans [46], and TM7 were also abundant in a transmissible colitogenic microbiota in inflammasome-deficient mice [26]. The two other most prominent components of that colitogenic community were two phylotypes of the family *Prevotellaceae* [26]. The only OTU of this family in the present dataset was lacking in all MHH2009 samples and occurred significantly more abundantly in the MIT caeca compared to those of the MHH2011 mice (Table S3).

Another noteworthy MIT-specific OTU was the one identified as *Bilophila wadsworthia*. While this species occurs in the intestinal microbiota of healthy humans [47], it can also act as an opportunistic intestinal pathogen, for example the organism is present in human cases of appendicitis [48]–[50]. Intestinal blooms of *B. wadsworthia*, which can be induced by dietary interventions (saturated fatty acid-rich diets such as enhanced in milk- or other animal-derived fat), can lead to significantly increased colitis in both mono-associated and SPF C57BL *Il10^−/−^* mice [51]. This might also be considered as one factor influencing microbiota between the MIT and MHH facilities, since MIT feed contained 5% porcine fat, while the MHH feed used in both the breeding and experimental facilities was virtually free from animal fat.

An additional microbial component that should be mentioned in the context of this model is *Lactobacillus reuteri*, which occurred with one OTU in our dataset. It was extremely rare in MHH mice – we identified a single sequence across all MHH samples – but was present in all MIT mice. *Lactobacillus* species are known to induce the production of both pro- and anti-inflammatory cytokines [52], and in vitro as well as in vivo effects tend to vary between strains [53]. In different experiments, *Lactobacillus* cocktails containing *L. reuteri* have been found to both stimulate immune response in piglets [54] and to reduce *H. hepaticus*-associated inflammation in *Il10*-deficient mice [40]. If newly introduced into *Lactobacillus*-free wt mice, L. *reuteri* alone can trigger non-pathological low-level inflammatory processes in the small intestine [55]. As shown by Whary *et al.* [23], co-colonization with *L. reuteri* alone can be sufficient to trigger the development of typhlocolitis in B6-*Il10^−/−^* mice infected with *H. hepaticus*.

Given these components of the MIT microbiota, IBD in MIT mice might be initiated by the combined action of *H. hepaticus* and the reasonably abundant *L. reuteri*. The initial inflammatory response in the lower bowel might be intensified by a bloom of the opportunistic pathogen *Bilophila wadsworthia* and potentially further exacerbated by a combined expansion of the *Prevotellaceae* and TM7 OTUs; the combination of these bacteria leading to a colitogenic state of the microbiota.

This study of the intestinal microbiota composition of mice with differential susceptibility to *H. hepaticus*-induced colitis permits the generation of a testable hypotheses; which of the microbial components that might be responsible for increasing inflammation in MIT mice or dampening inflammation in MHH mice? For example, future studies should verify the roles of both *L. reuteri* and *B. wadsworthia* in colitis development, and investigate whether the presence of one or both of these species in non-rare numbers in combination with the MHH microbiota would lead to colitis development during *H. hepaticus* infection.

## Materials and Methods

### Mice

We analyzed the intestinal microbiota composition of three groups of mice that were not infected with *H. hepaticus*. Each group consisted of 8 C57BL/6J *Il10^−/−^* (B6.129P2-*Il10^tm1Cgn^*/J or B6.129P2-*Il10^tm1Cg^*/JZtm, respectively) mice kept under SPF conditions. Two of these groups (MHH2009 and MHH2011) were housed at Hannover Medical School (MHH), and one group was housed at Massachusetts Institute of Technology (MIT). MHH animals were derived from a nucleus colony regularly screened during routine genetic monitoring [56]. The MHH2009 group consisted of mice sacrificed at 38 (4 mice) or 141 (4 mice) days of age (MHH2009). MIT mice were necropsied at 172 days of age, and MHH2011 mice at 204 days of age. MHH2009 and MHH2011 mice were from the same breeding colony used as a source of mice for *H. hepaticus* infection experiments, both groups were necropsied approximately 16 months apart. From the colocecal junction of each MHH2009 and MIT animal, we obtained one colon and one cecum sample. From MHH2011 animals, only cecum samples were included. The samples were snap-frozen in liquid nitrogen before being placed at −80°C.

### *H. hepaticus* Infections

*H. hepaticus* infections with the wild type strain ATCC 51449 (3B1) were performed and monitored exactly as described previously [17]. Infections at MHH and MIT were performed with the same *H. hepaticus* strain, and a *H. hepaticus* strain exchange between MIT and MHH was performed to rule out that differences between strains were the reason for the observed differences in pathology. C57BL/6J *Il10^−/−^* mice were bred at the animal facility of Hannover Medical School under *Helicobacter*-free SPF conditions. Access to mice was limited to few animal caretakers who had to pass through a water shower and were required to wear a gown, cap, surgical mask, overshoes, and gloves. Mice were housed separated by sex in open cages (540 cm^2^ floor area) at a maximum of five animals on bedding of autoclaved, dust-free, softwood fibers. Sterile pelleted diet (ssniff R-Z, ingredients include vegetable protein and fat, but no animal-derived ingredients) and deionized, filtered, and UV light-treated water were provided ad libitum. The breeding colony was regularly monitored for the presence of common murine pathogens and various *Helicobacter* species according to FELASA recommendations [57], and all animals were tested again for the presence of *Helicobacter* species before the start of an experiment. For infection experiments, animals were transferred to an S2 infection unit, where animals were kept in ventilated isolator cages under SPF conditions and fed sterile water and irradiated chow (ssniff M-Z feed; ingredients include soy protein and vegetable fat, but no animal-derived ingredients) ad libitum. All experiments at MHH involving mice were conducted in accordance with the German animal protection law and with the European Communities Council Directives 86/609/EEC and 2010/63/EU for the protection of animals used for experimental purposes. All experiments at MHH were approved by the MHH Animal Protection Officer (“Tierschutzbeauftragter der MHH”) and permitted by the local authority (Lower Saxony State Office for Consumer Protection, Food Safety, and Animal Welfare Service, AZ 33.9-42502-04-08/1456). At MIT, *Il10* ^−/−^ C57BL/6J mice (Jackson Laboratories, Bar Harbor, ME) were maintained free of known murine viruses, *Salmonella spp., Citrobacter rodentium*, ecto- and endoparasites, and known *Helicobacter spp*. in a facility accredited by the Association for Assessment and Accreditation of Laboratory Care International, under barrier conditions. All experiments performed at MIT were approved by the “MIT Committee on Animal Care”. Animals were housed in microisolator, solid-bottomed polycarbonate cages on hardwood bedding, fed a commercial pelleted diet (Prolab RMH 3000; ingredients include fish meal and 5% porcine fat conserved with BHA), and administered water ad libitum. The protocol was approved by the MIT Committee on Animal Care.

To monitor the infection status throughout the experiment, fecal pellets were collected once a week and the presence or absence of *H. hepaticus* was assessed by semi quantitative PCR from total DNA isolated from the stools (Tissue Amp Kit, Qiagen Inc.). Usually, no weight loss or any other external signs of disease or ill health upon visual inspection of the animals were observed throughout the observation period. Occasionally, loose stools were observed with the infected animals.

After necropsy, tissue samples from both cecum and colon were weighed, homogenized and plated on selective blood plates for *H. hepaticus* cfu counts per mg tissue (MHH), or DNA was extracted for quantitative PCR (MIT) [58], [59]. CFU counts from MHH infection experiments and qPCR-derived *H. hepaticus* genome counts from the corresponding MIT infections yielded similar results. At MHH, *H. hepaticus* 3B1 infection of mice from the same breeding colony and born within 6 days of the MHH2011 animals resulted in 5.5 * 10^7^ to 6.1 * 10^8^ cfu per g cecum. At MIT, the qPCR analysis of cecal samples from 3B1-infected mice yielded 10^3^ to 10^6^ genome copies per µg mouse DNA, corresponding to 10^5^–10^8^ bacteria per g tissue [20]. Samples from the cecum and colon were also fixed and embedded for tissue sectioning and pathological scoring.

### Mouse Pathology

Tissue sections were scored according to the scoring system developed by Burich *et al.* [29] by blinded pathologists who are experts in rodent intestinal pathology. To obtain the direct comparison shown in Fig. 1, unstained slides of samples from both facilities were exchanged and read by one pathologist who was blind to the sample identity.

### DNA Extraction

DNA was extracted using the QIAamp DNA Mini Kit (Qiagen). Samples were lysed overnight in Buffer ATL supplemented with Proteinase K (both Qiagen) at 56°C, and DNA was extracted according to the QIAamp DNA Mini Kit tissue protocol. DNA was quantified on a NanoDrop 1000 spectrophotometer (Thermo Scientific).

### Partial Amplification and Deep Sequencing of 16S rRNA Genes

For MHH2009 and MIT samples, 16S V2 amplicons for 454 pyrosequencing were prepared as described by Turnbaugh *et al.* [60]. 16S ribosomal DNA gene fragments were amplified using 454 FLX fusion primers containing 454 adapter sequences, linker nucleotides, multiplex identifiers (MIDs) and template-specific parts consisting of the universal bacterial primers 8F (5′- AGAGTTTGATCCTGGCTCAG-3′) and 338R (5′-TGCTGCCTCCCGTAGGAGT-3′). PCR reactions contained 30–50 ng DNA, 0.3 µM of each primer, 2.5 mM Mg^2+^, 0.2 mM of each dNTP, and 2.5 U HotMaster Taq Polymerase (5PRIME) per 50 µl reaction. Cycling conditions were 95°C for 2 min followed by 30 cycles of 20 s at 95°C, 20 s at 52°C, and 1 min at 65°C. PCRs included a positive control and a no-template negative control, and PCR success was confirmed on 1% agarose gels. Amplicons were purified with AMPure beads (Agencourt, Beckman Coulter) as described in the 454 FLX User Manual and quantified with a NanoDrop spectrophotometer. Length and amplicon integrity were controlled with Agilent DNA 1000 Chips on a 2100 Expert Bioanalyzer (Agilent). Sets of 8 samples with different MIDs were pooled at 200 ng per sample. Emulsion PCR was carried out and samples were sequenced from the 3′ end according to the 454 FLX manual. Raw data were processed with the amplicon pipeline of GS Run Processing Software version 2.3 (454 Life Sciences) using default settings.

For the MHH2011 cecum samples, 16S ribosomal DNA gene fragments were amplified as described in Lofgren *et al.* [61] 454 FLX Titanium fusion primers included MIDs and template-specific parts consisting of the universal bacterial primers 8F (5′-AGAGTTTGATCCTGGCTCAG-3′) and 541R (5′-WTTACCGCGGCTGCTGG-3′). The primer pair was tested for compatibility with primer pair 8F-338R using the SILVA database tool TestPrime (v. 1.0, database SSU r114 RefNR) [62] in combination with custom vba and sql code (Table S5). The broad agreement between the sets of sequences matching each primer pair indicated that the differences between the microbiota of MHH2011 mice on the one hand and MIT and MHH2009 mice on the other hand are unlikely to be due to primer differences. PCR reactions contained 30 ng DNA, 0.3 µM of each primer, 2.5 mM Mg^2+^, 0.2 mM of each dNTP, and 2.5 U HotMaster Taq Polymerase (5PRIME) per 50 µl reaction. Cycling conditions were adapted following the 454 FLX Titanium manual: 95°C for 2 min followed by 30 cycles of 30 s at 94°C, 30 s at 60°C, and 80 s at 72°C. PCRs included a positive control and a no-template negative control. PCR products were run on 1% agarose gels, and 650–700 bp fragments were extracted using the QIAquick Gel extraction kit (Qiagen). Extracted amplicons were quantified using the Quant-iT PicoGreen dsDNA Kit (Invitrogen) on a TBS-380 Fluorometer (Turner BioSystems). Sets of 8 samples with different MIDs were pooled at 40 ng per sample. Emulsion PCR was carried out using the emPCR Titanium Lib-L kit. Samples were sequenced from the 3′ end using the Titanium Sequencing Kit XLR70 according to the 454 FLX Titanium manual (version of 20^th^ October 2009). Raw data were processed with the amplicon pipeline of GS Run Processing Software version 2.3 as above, but with the TrimBackScaleFactor set to 2∶160. Sequences were submitted to the European Nucleotide Archive (ENA) under project accession number PRJEB4164 (secondary study accession number ERP003425).

#### Sequence processing and bioinformatic analysis

Sequences were pre-processed using the software package mothur [30] (Linux 32-bit version 1.22.1 and Windows 32-bit version 1.21.1) following the suggestions in the “Schloss SOP” tutorial [63], [64]. Briefly, primers and barcodes were removed, and sequences were trimmed where the average quality score over a 50-bp-window dropped below 35. Sequences were considered low quality and culled if they contained homopolymer stretches longer than 8 bp, included ambiguous base calls, or were shorter than 150 bases. Remaining non-identical sequences were aligned to the “SILVA” reference alignment [65] with mothur’s align.seqs algorithm, using the default settings of 8-mer searching and Needleman-Wunsch alignment. Sequences which did not align in the expected region of the reference alignment were removed. PCR chimeras were identified using UCHIME [66] with the mothur-provided “SILVA gold” reference set [65], and culled.

The resulting dataset was analyzed with Ribosomal Database Project (RDP) classifier version 2.4 with the hierarchy model of training data no. 7 [31]. Sequences were classified to the lowest taxonomic rank that received at least 70% bootstrap support (BTS). Following Ochman *et al.* [32], sequences which could not be assigned to class at BTS 70% were excluded from further analyses, except when they were classified to lower taxonomic ranks for which no class was designated. Potential organellar sequences were further analyzed by blastn similarity search against the SILVA reference database, build 108 [67]. In order to obtain the possibility to reliably identify sequences to species, the RDP 16S rRNA database (release 10, update 28, matching RDP classifier training data no. 7) was modified using TaxCollector [68] to include taxonomic descriptions with easily identifiable species designations. Query sequences identified to genus level by RDP classifier at a stringent cutoff of 97% BTS were searched against this TaxCollector-modified RDP database using MegaBlast [69]. Sequences were considered as annotated to species level if their identity to the best hit in the database was at least 97%, the aligned region covered at least 97% of the query sequence length, they did not match a second species at the same BLAST Expectation value, and the identified species did not conflict with the genus as identified by RDP classifier.

In order to facilitate comparison of these data with the altered Schaedler flora, the sequences provided by Dewhirst *et al.* [33] were similarly analyzed by RDP classifier.

OTUs were calculated using ESPRIT-Tree [70]. In order to avoid artifacts due to the inclusion of the additional sequence stretch in the longer MHH2011 sequences, all sequences were first trimmed to the region corresponding to the 8F-338R amplicon as identified in the mothur-generated alignment. Following Cai and Sun [70], the cutoff OTU level to best represent species level was determined using the normalized mutual information (NMI) criterion [71] on a subset of sequences that could be identified to species. For sequences classified to species, the NMI between species and OTU classification was calculated following Fred and Jain [71]. After comparing NMI values for OTUs obtained for 30 distance levels from 0.01 to 0.3 (Fig. S1), we retained the OTUs at level 0.08, which resulted in peak NMI values. To link all sequences in the dataset with both OTU and taxonomic information, OTUs were classified to the lowest possible rank based on the 80% consensus of their respective RDP classifications using a custom VBA script. Additionally, OTU consensus sequences were computed in Geneious Basic v. 5.6.4 [72] based on alignments generated using MUSCLE [73]. For alignment of OTU 0.08_group_279, which contained too many sequences to be computed in MUSCLE, we used MAFFT [74] with the settings “FFT-NS-2–ep 0.123”. Consensus sequences were classified using the RDP classifier and the TaxCollector-modified RDP database as detailed above. As OTU consensus sequences could often be classified to a lower taxonomic rank than the majority of their component sequences, but were only crudely classified if they contained ambiguous bases, we chose the more detailed of the two classifications for each OTU (Table S6).

To further analyze and visualize the final dataset, a mothur-compatible “sharedfile” was reconstructed from the OTU information. The Windows version of mothur (version 1.21.1) was used for a range of statistical and visualization steps: To avoid artifacts produced by unequal sample sizes, the sequences from each mouse sample were subsampled to match the 1227 sequences found in the sample with the lowest sequence count. Rarefaction curves and rank-abundance-plots were generated. Differences among samples were visualized by Principal Coordinates Analysis (PCoA) using Jaccard index distances, which measure dissimilarity of microbiome composition. Mothur was also used to generate Venn diagrams (see below).

A heatmap of OTU abundances among samples was produced using the R script heatmap [75]. Input data for heatmap construction was based on the mothur-type sharedfile, which was transformed to facilitate visualization of the full range of OTU abundances. This transformation was carried out according to the formula h = LN(count+0.1), where h is the input value for the heatmap script, count is the sequence count for one OTU in an individual sample, LN is natural logarithm and 0.1 is used as a small increment to avoid occurrences of LN(0). The heatmap color key was manually modified to correspond to untransformed OTU abundances. To increase readability, individual OTU labels were replaced with taxonomic information and sample labels were replaced with sample numbers and group information using Inkscape 0.48.

Sequences which did not occur in at least 4 samples of either the cecum or the colon samples of at least one mouse group were classified as too rare to judge site specificity and excluded from the lists of site-specific OTUs and from the Venn diagrams. For additional comparisons, significance of abundance differences between sample groups was assessed using Metastats [76], as implemented in the original R script, at a significance cutoff of p≤0.05.

## Supporting Information

Figure S1 **Normalized mutual information (NMI) value between species and OTUs obtained for 30 difference levels using ESPRIT-Tree.** NMI = 1 would indicate complete agreement between species and OTU boundaries for all sequences analyzed. For this dataset, NMI peaks at OTU cutoff 0.08 and an NMI score of 0.98.
(DOC)

Table S1 **Genera as identified by RDP classifier, including distribution among individual samples.**
(XLS)

Table S2 **Distribution of OTUs among individual samples after subsampling to 1227 sequences per sample, including OTU classification.**
(XLS)

Table S3 **OTU overview, including OTU classification, aggregate information on OTU distribution among sample sets, and results of Metastats comparisons between sample sets (p< = 0.05).**
(XLS)

Table S4 **List of Schaedler Flora sequences classified according to the criteria also employed for the experimental sequences.**
(XLS)

Table S5 **Comparison of primer pairs 8F-338R and 8F-541R according to SILVA TestPrime results.** Included groups correspond to the taxonomic identification of OTUs identified as restricted to one animal facility or significantly more abundant in one set of samples.
(XLS)

Table S6 **OTU classification according to (1) 80% consensus of classification of individual sequences and (2) classification of OTU consensus sequences (3) final OTU classification.**
(XLS)
