# Supplementary material for: Intestinal Microbiota Composition of Interleukin-10 Deficient C57BL/6J Mice and Susceptibility to Helicobacter hepaticus-Induced Colitis
Source: PLoS One. 2013 Aug 9;8(8):e70783. doi: 10.1371/journal.pone.0070783 (PMC3739778; doi:10.1371/journal.pone.0070783)
Supplement: Figure S1 — Normalized mutual information (NMI) value between species and OTUs obtained for 30 difference levels using ESPRIT-Tree. NMI = 1 would indicate complete agreement between species and OTU boundaries for all sequences analyzed. For this dataset, NMI peaks at OTU cutoff 0.08 and an NMI score of 0.98. (DOC) [file pone.0070783.s001.doc]

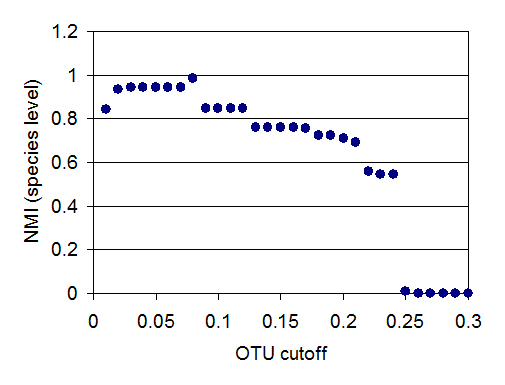


Fig. S1

Normalised mutual information (NMI) value between species and OTUs obtained for 30 difference levels using ESPRIT-tree. NMI=1 would indicate complete agreement between species and OTU boundaries for all sequences analysed. For this dataset, NMI peaks at OTU cutoff 0.08 and an NMI score of 0.98.
